# Effect of Propolis on Experimental Cutaneous Wound Healing in Dogs

**DOI:** 10.1155/2015/672643

**Published:** 2015-12-13

**Authors:** Ashraf M. Abu-Seida

**Affiliations:** Department of Surgery, Anesthesiology & Radiology, Faculty of Veterinary Medicine, Cairo University, Giza 12211, Egypt

## Abstract

This study evaluates clinically the effect of propolis paste on healing of cutaneous wound in dogs. Under general anesthesia and complete aseptic conditions, two full thickness skin wounds (3 cm diameter) were created in each side of the chest in five dogs, one dorsal and one ventral, with 10 cm between them. These wounds were randomly allocated into two groups, control group (10 wounds) and propolis group (10 wounds). Both groups were represented in each dog. The wounds were cleaned with normal saline solution and dressed with macrogol ointment in control group and propolis paste in propolis group, twice daily till complete wound healing. Measurement of the wound area (cm^2^) was monitored planimetrically at 0, 7, 14, 21, 28, and 35 days after injury. The data were analyzed statistically. The results revealed a significant reduction in the wound surface area in the propolis group after 14 and 21 days compared to control group. The wound reepithelization, contraction, and total wound healing were faster in propolis group than in control group during five weeks of study. In conclusion, propolis paste has a positive impact on cutaneous wound healing and it may be suggested for treating various types of wounds in animals.

## 1. Introduction

Wound healing results from a complex tissue repairing process to replace devitalized and missing cellular structures and tissue layers. This process is divided into four precisely and highly programmed phases including blood clotting, inflammation, the growth of new tissue (proliferation), and the remodeling of tissue (maturation). Several factors such as age, sex, nutrition, stress, infection, and medication can interfere with one or more phases of this process, thus causing improper or impaired wound healing. Although several wound healing agents are used in veterinary practice, new agents are usually discovered [1].

Propolis is a resinous material collected by bees from plants exudates and buds and mixed with wax and bee enzymes. It consisted of 30% beeswax, 50% resins and vegetable balsams, 10% essential oils, 5% pollen, and 5% other substances. Its color varies from green and red to dark brown. Propolis has a characteristic smell and shows adhesive properties because it strongly interacts with oils and proteins of the skin [2]. In contrast, geopropolis is produced by indigenous stingless bees and it is composed of resinous material of plants and soil or clay [3].

Etymologically, the Greek word propolis means* pro*, for or in defense, and* polis*, the city, that is, “defense of the hive.” Propolis is a multifunctional material used by bees in the construction and maintenance of their hives. Bees use it to seal holes in their honeycombs, smooth out internal walls, and cover carcasses of intruders who died inside the hive in order to avoid their decomposition. Propolis also protects the colony from diseases because of its antiseptic efficacy and antimicrobial properties [4].

The use of propolis goes back to ancient times, at least to 300 BC, and it has been used as a medicine for internal and external uses in many parts of the world. Egyptians, Greeks, and Romans reported the use of propolis for its general healing qualities. Ancient Egyptians used it to embalm their dead, and more recently it was used during the Boer War for healing [5].

Propolis has been used empirically for centuries and it has several biological applications including acceleration of regenerating processes in the damaged cartilages and bones [6, 7], immunomodulatory [8], antimicrobial [9], antioxidant [10], analgesic, and anti-inflammatory agent [11], and antitumoral property [12]. Since propolis possesses these several biological properties, this study aims to evaluate clinically the potential therapeutic effects of propolis on cutaneous wound healing in dogs.

## 2. Materials and Methods

### 2.1. Animals

This study was approved by the Animal Use and Care Committee at Faculty of Veterinary Medicine, Cairo University, Egypt. All surgeries were performed under general anesthesia, and all efforts were made to minimize animal suffering and to reduce the number of animals used.

A total of five adult mongrel dogs (3 males and 2 females) aged approximately 1-2 years and weighing 20–25 kg were selected for this study. The animals were housed under standard environmental conditions (23 ± 1°C, with 55 ± 5% humidity and a 12 h light/dark cycle) and maintained with free access to water and three meals per day, including dry food (Sportmix-Adult, USA) and milk.

### 2.2. Preparation of MP (Carrier)

To prepare 100 g of Macrogol ointment, 40 g of polyethylene glycol 3350 (Ineos Manufacturing, Deutschland GmbH, Germany) was mixed with 60 g of polyethylene glycol 400 (DOW Chemical Company, USA). The two ingredients were heated in water bath at 65°C until complete melting and then allowed to cool down to room temperature while stirring until the mixture was congealed.

### 2.3. Formation of the Propolis Paste

To prepare 50 g of propolis paste, 15 g of propolis (Bee Propolis, Holistic Herbal Solutions, LLC, USA) was mixed well with 35 g of MP in a sterile mortar to obtain a creamy paste.

### 2.4. Creation of Skin Wounds

All dogs were premedicated with subcutaneous injection of atropine sulphate (Atropine, ADWIA, Egypt) at a dose of 0.05 mg/kg body weight and intravenous injection of xylazine HCl (Xylazine 2%, Alfasan, Belgium) 1 mg/kg body weight as a premedication. The dogs were generally anesthetized by using ketamine HCl (Ketamine 5%, TRITTAU, Germany) 5 mg/kg body weight given I.V. via a 20-gauge cannula. Then the general anesthesia was maintained by 25 mg/kg incremental doses of 2.5% solution of thiopental sodium (Thiopental Sodium, EPICO, Egypt).

Under complete aseptic conditions, four circular full thickness skin wounds (3 cm diameter) were created at both sides of the chest in each dog (2 wounds in each side, one dorsal and one ventral, with 10 cm between them). These wounds were left open to heal by the secondary intention. All dogs were given intramuscular cefotaxime sodium at a dose of 10 mg kg and diclofenac sodium at a dose of 1.1 mg kg once/day for 5 days after surgery for pain and infection control [13]. These wounds were divided randomly into two equal groups (*n* = 10) including group I (control group) and group II (treated group). In control group, the wounds were cleaned with normal saline solution and dressed with macrogol ointment twice daily until complete wound healing. In treated group, the wounds were cleaned with normal saline solution and then dressed with 1 mL propolis paste twice daily. Measurement of the wound area (cm^2^) was monitored planimetrically at 0, 7, 14, 21, 28, and 35 days after injury (DAI) according to the method described by Oryan et al. [14]. The measurement was carried out by another veterinarian who was blinded to the experimental design and group allocation.

### 2.5. Statistical Analysis

The data were expressed as mean and standard deviation. The one-way ANOVA followed by Turkey post hoc test were used for comparison of different parameters. The data were analyzed by SPSS software, version 16.0 (SPSS Inc., Chicago, IL, USA), and *P* ≤ 0.05 was accepted as statistically significant.

## 3. Results

The surface area of all wounds was calculated and expressed in cm^2^ as shown in Table 1.

**Figure 1:**
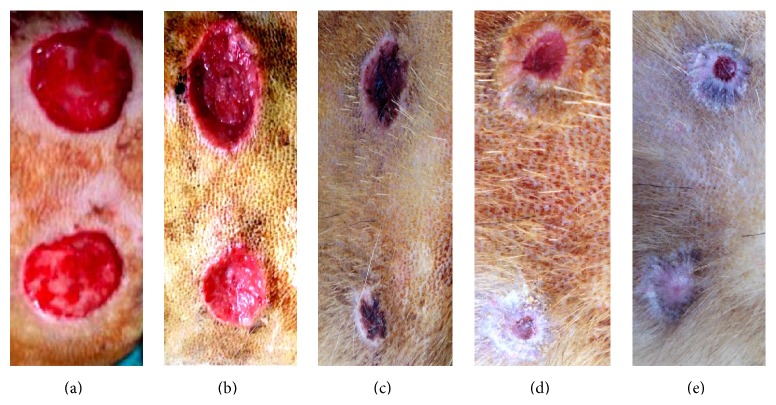
Representative skin wounds of the control group (upper wounds) and propolis group (lower wounds) at 0 (a), 7 (b), 14 (c), 21 (d), and 28 (e) days after surgery.

The follow-up of wounds treated with propolis paste showed wound healing started at day three after injury and evident healing after seven days with significant difference compared to untreated wounds until the end of the study. The centre of the treated wounds became a scar and the total wound size appeared lesser than those of control group along the duration of the study. There was a significant reduction in the wound surface area in the propolis group on days 14 and 21 compared to those in the control group (Figure 1).

The wound reepithelization, contraction, and total wound healing were faster in propolis treated group than in control group during five weeks of study. In addition, no side effects were recorded after application of propolis paste during this study.

## 4. Discussion

Nonhealing wounds and wounds with secondary infection by multidrug resistant bacteria are a common challenge in veterinary practice. Therefore, several recent studies have been conducted to investigate various synthetic and biomaterials for enhancing wound healing in both humans and animals. These agents included glycerol, tripeptide copper complex (TCC) hydrogel, platelet rich plasma (PRP), zinc compounds,* Aloe vera*, sildenafil, tocopherol, pomegranate (*Punica granatum*),* Lantana* (*Lantana camara*), chitosan, stem cell therapy, honey, and hydroethanolic extract of ribwort plantain leaves [15, 16].

Cutaneous wound healing is a complex process involving the interplay of various cell types in the injured tissue, including inflammatory cells, keratinocytes, fibroblasts, and endothelial cells [17]. Wound healing is a natural body reaction initiated immediately after injury and occurs in four stages including coagulation, inflammation, reepithelization, and remodeling [18].

The skin is usually subjected to many injuries including chronic unhealed wounds, burns, and ulcers which do not heal at all or do so very slowly [19]. Moreover, acceleration of the healing of acute wounds is also required. The nonhealing wounds are a large and growing problem, so several treatments were applied to enhance the wound healing, but wounds do not respond well to many of them. Therefore, there is a continuous need to develop agents that accelerate the healing of acute and chronic wounds and ulcers and regenerate of damaged tissue in burns.

The ideal topical wound treatment product must be biocompatible, nontoxic, and able to enhance the healing without adverse effects on the progress of the natural wound healing process [20]. To date, there is no single optimal treatment that enhances the resolution of problem wounds [21].

In the last decades, propolis has attracted researchers' interest because of several biological and pharmacological properties [22]. Therefore, propolis, as a natural product with useful biological properties and no recorded side effects, was selected in the present study to evaluate clinically its effect on healing of full thickness skin wound.

As regards the chemical composition of the used propolis in this study, the major constituents of propolis were flavonoids which contribute greatly to the pharmacological activities of propolis. Flavonoids have a broad spectrum of biological activities, such as anti-inflammatory, antibacterial, and antiviral effects. Moreover, the used propolis had phenylpropanoids, terpenoids, stilbenes, lignans, coumarins, and their prenylated derivatives. Terpenoids represented 10% of the propolis constituents and they exhibit antioxidant and antimicrobial effects. In addition, the used propolis had large amounts of caffeic acid phenethyl ester (CAPE), 3-methyl-but-2-enyl caffeate, isopentyl ferulate, and moronic acid. The broad spectrum of biological properties of propolis is attributed to the variety of these major chemical constituents.

The present data showed that propolis helps the wound healing in a time-dependent manner. This could be attributed to immunomodulatory [8], antimicrobial [9], antioxidant [10], analgesic, and anti-inflammatory [11] effects of the propolis. These biological effects are essential for acceleration of the wound healing process. Caffeic acid phenethyl ester (CAPE) derived from the propolis has immunosuppressive activity in T-cells which play a key role in the onset of several inflammatory diseases. Moreover, CAPE specifically inhibited both interleukin- (IL-) 2 gene transcription and IL-2 synthesis in stimulated T-cells [23]. Data suggest an increase in the fungicidal activity of macrophages by propolis. In addition, propolis inhibits bacterial growth by preventing cell division, disorganizing the cytoplasm, the cytoplasmic membrane, and the cell wall, causing a partial bacteriolysis and inhibition of protein synthesis. Moreover, propolis contains 3-methyl-but-2-enyl caffeate, isopentyl ferulate, and moronic acid which have a significant antiviral activity [23].

In the present study, propolis paste was applied twice daily because it strongly interacts with oils and proteins of the skin resulting in good adhesion and prolonged action. Similarly, Burdock [2] mentioned that the use of products containing propolis has resulted in extensive dermal contact.

The results of this study demonstrated that topical application of propolis paste 30% enhanced wound contraction and reduced the healing time. In addition to its safety and effectiveness, propolis is an inexpensive topical wound treatment natural product. Therefore, propolis could be considered as a good alternative to several synthetic topical wound treatment products.

## 5. Conclusion

In conclusion, propolis paste 30% has a positive impact on cutaneous wound healing and it may be suggested for treating various types of wounds in animals.

## Figures and Tables

**Table 1:** Mean ± SD of wound surface area (cm^2^) in both groups on different days after injury.

Days	Control group	Propolis group
7	5.54 ± 0.27	4.9 ± 0.36
14	3.38 ± 0.41^a^	2.1 ± 0.27^b^
21	1.1 ± 0.2^a^	0.36 ± 0.1^b^
28	0.28 ± 11	0.0 ± 0.0
35	0.0 ± 0.0	0.0 ± 0.0

Different letters in the same row are statistically significantly different at *P* ≤ 0.05.
